# The cellular prion protein mediates Tau oligomer‐induced memory impairment in mice

**DOI:** 10.1002/alz.092149

**Published:** 2025-01-03

**Authors:** Gianluigi Forloni, Claudia Balducci, Franca Orsini, Milica Cerovic, Antonio Masone, Ilaria Raimondi, Giada Lavigna, Chiara Zucchelli, Marten Beeg, Laura Colombo, Giovanna Musco, Marco Gobbi, Roberto Chiesa, Luana Fioriti

**Affiliations:** ^1^ Istituto di Ricerche Farmacologiche Mario Negri IRCCS, Milano Italy; ^2^ IRCCS Ospedale S. Raffaele, Milano Italy

## Abstract

**Background:**

The role of oligomeric forms of various proteins as direct responsible of neuronal dysfunction in neurodegenerative disorders has been supported by numerous findings at experimental level and, more recently, by histological examinations in human material. The cellular prion protein (PrP^C^) has been proposed to mediate the neurotoxicity of β‐amyloid, α‐synuclein and tau oligomers. We demonstrated that although amyloid‐β oligomers (AβOs) bind with high affinity to PrP^C^, the memory deficit induced by intracerebroventricular (ICV) administration of AβOs in mice was not mediated by PrP^C^. Moreover, we did not confirm the reported interaction between α‐synuclein oligomers and PrP^C^, and found that their effect on memory did not depend on PrP^C^ expression. In this study, we examined the interactions between tau oligomers (TauOs) and PrP^C^ by chemico‐physical and functional studies.

**Method:**

TauOs were prepared in the presence of arachidonic acid and analyzed by atomic force microscopy; the interaction between TauOs and PrP^C^ was investigated by surface plasmon resonance (SPR); the cellular localization of TauOs was studied in HEK293 cells expressing different levels of PrP^C^; the effect of TauOs on memory function was assessed by the novel object recognition test (NORT) after ICV injection (1µM 7.5 µl) in wild type (wt) and PrP knockout (KO) mice; the effect of TauOs on long‐term potentiation (LTP) was analyzed in hippocampal slices.

**Result:**

SPR demonstrated a high‐affinity binding between TauOs and PrP^C^ with a Kd in the range of 20‐50 nM. Immunofluoresce analysis showed a PrP^C^ dose‐dependent association of TauOs with the plasma membrane of HEK293 cells, and their co‐localization with PrP^C^. ICV application of TauOs induced memory impairment in wt but not in PrP KO mice. Accordingly, TauOs inhibited hippocampal LTP in a PrP^C^‐dependent fashion.

**Conclusion:**

In contrast to our previous findings with Aβ and α‐synuclein oligomers, we demonstrate that PrP^C^ interacts with TauOs and this binding has functional consequences. This interaction might represent an interesting therapeutic target in Alzheimer’s disease and other tauopathies.

